# Leisure-Time Physical Activity in Subjects with Metabolic-Dysfunction-Associated Steatotic Liver Disease: An All-Cause Mortality Study

**DOI:** 10.3390/jcm13133772

**Published:** 2024-06-27

**Authors:** Ritanna Curci, Caterina Bonfiglio, Isabella Franco, Claudia Beatrice Bagnato, Nicola Verrelli, Antonella Bianco

**Affiliations:** 1 Laboratory of Movement and Wellness, National Institute of Gastroenterology, IRCCS “S. de Bellis”, Via Turi, 70013 Castellana Grotte, BA, Italy; ritanna.curci@irccsdebellis.it (R.C.); isabella.franco@irccsdebellis.it (I.F.); claudia.bagnato@irccsdebellis.it (C.B.B.); nicola.verrelli@irccsdebellis.it (N.V.); 2 Laboratory of Epidemiology and Statistics, National Institute of Gastroenterology, IRCCS “S. de Bellis”, 70013 Castellana Grotte, BA, Italy; catia.bonfiglio@irccsdebellis.it

**Keywords:** physical activity, metabolic-dysfunction-associated steatotic liver disease, mortality, leisure time

## Abstract

**Background:** Metabolic-dysfunction-associated steatotic liver disease (MASLD) affects 30% of adults worldwide and is associated with obesity and cardiovascular risk factors. If left untreated, it can progress to severe liver disease. Lifestyle changes such as physical activity and weight loss help to reduce the severity and risk of mortality. This study estimated the impact of MASLD and leisure-time physical activity (LTPA) on mortality and examined how gender mediates this effect in a Southern Italian population. **Methods:** This work is a population-based prospective cohort study of inhabitants of Castellana Grotte (>30 years old) in Southern Italy, which began in 1985. Participants provided general health information, underwent anthropometric measurements and ultrasonography, and completed a validated questionnaire on their food intake and LTPA. The vital status was tracked through local municipalities **Results:** In total, 1826 participants (39% with MASLD) were enrolled in this study, drawn from 2970 eligible subjects; the mean age was 51.91 (±14.76) years and 56.2% were men. Subjects with MASLD who practiced low LTPA had a significantly higher risk of death than those who did not have MASLD and practiced high LTPA. In addition, subjects with MASLD who practiced low LTPA were about 19% less likely to survive to the age of 82 years. As regards gender, both men and women with MASLD and low LTPA showed a significant risk of death, but this was higher in women. **Conclusions:** The presence of MASLD, especially in women, increases the risk of death from all causes. LTPA plays a key role in the disease and reduces mortality in these individuals.

## 1. Introduction

Metabolic-dysfunction-associated steatotic liver disease (MASLD) affects about 30% of the adult world population. It is considered one of the most common chronic liver diseases, showing a sharp increase over the past 20 years. Its prevalence parallels that of obesity and related diseases [1].

The most recent definition of MASLD was published in June 2023 by a multi-society Delphi consensus statement; it is considered to be more inclusive and less stigmatizing and reduces the exclusion criteria compared to the previous definitions of metabolic-associated fatty liver disease (MAFLD) and, before this, non-alcoholic fatty liver disease (NAFLD) [2]. The diagnostic criteria have also been changed, so that MASLD can be diagnosed in patients with hepatic steatosis and one of five cardiovascular risk factors [3]. 

MASLD is a disease characterized by excessive fat in the liver exceeding 5%, verified by liver imaging or biopsy [4]. It is associated with cardiovascular risk factors, which play a key role in the development and severity of MASLD, including obesity, sedentary behavior and little exercise, the influence of genetics, and environmental factors. They contribute to the development of insulin resistance, oxidative stress, inflammation, and mitochondrial dysfunction [2]. MASLD’s signs range from simple steatosis to steatohepatitis, which can progress to liver fibrosis and cirrhosis, as well as hepatocellular carcinoma [5]. 

A nonpharmacological approach including nutritional interventions and regular physical activity (PA) may be the optimal strategy to manage MASLD and reduce its severity [6]. Weight loss by about 10% produces beneficial effects in individuals with MASLD, reducing its severity; likewise, PA can reduce steatosis and manage risk factors [7]. 

Li [8] studied the correlation between PA and MASLD and found that leisure-time physical activity (LTPA) was crucial for the management and reduction of MASLD. Specifically, by dividing PA into different categories, it was seen that LTPA had a greater impact than the others, and increased activity was associated with a reduction in the likelihood of contracting MASLD [8]. A large body of scientific evidence has shown the inverse association between PA and all-cause mortality in individuals with NAFLD. Specifically, it has been shown that high daily PA levels are associated with reduced all-cause mortality in adults with NAFLD [9]. Moreover, subjects who performed more LTPA had longer long-term survival than inactive subjects [10].

Several studies conducted by the Laboratory of Epidemiology and Biostatistics of the IRCCS Saverio de Bellis have highlighted the effectiveness of different physical activity programs for NAFLD, including aerobic and endurance sessions. Recently, we also showed that the probability of a certain degree of NAFLD severity is greater in women aged over 50 years old.

We then hypothesized that people with MASLD and low levels of PA could have a higher risk of death and that this effect on mortality may be partially mediated by gender.

This study aimed to estimate the effect of MASLD and different grades of PA on all-cause mortality and to quantify the extent to which gender mediates the effect from exposure to outcome, in a population-based study of people aged 30 years or older from Southern Italy, followed up with for 18 years.

## 2. Materials and Methods

Details of the study population have been published elsewhere [11]. Briefly, the MICOL study is a population-based prospective cohort study randomly drawn from the electoral rolls of Castellana Grotte (≥30 years) in 1985, with the participants followed up with over time with 3 follow-ups: (1992–1993; 2005–2006; 2017–2019). This work refers to the second follow-up (2005-06), when a random sample of subjects (PANEL study) aged 30–50 years was added to the initial cohort to compensate for the aging of the cohort.

**Figure 1:**
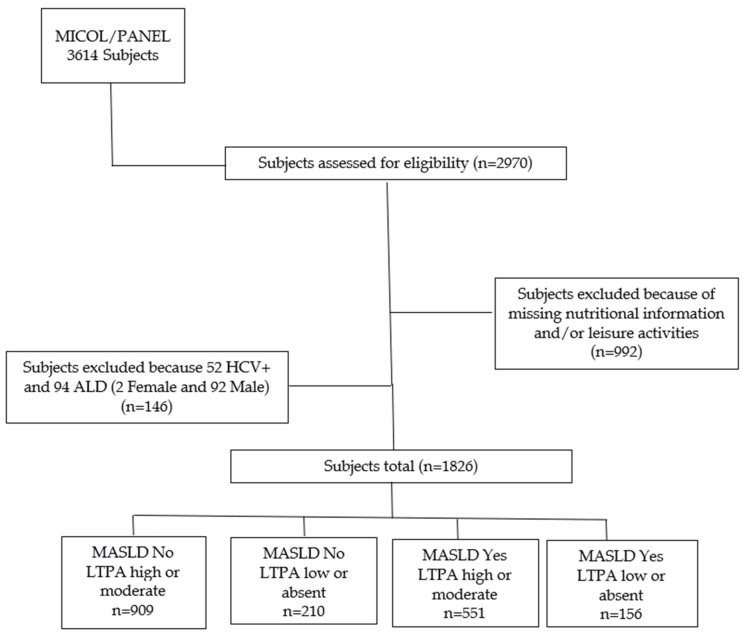
Flow chart. ALD: Alcohol-related Liver Disease; MASLD: Metabolic dysfunction-associated steatotic liver disease; LTPA: Leisure Time Physical Activity.

In total, 2970 eligible subjects out of 3614 (82.2% response rate) gave written informed consent to participate. Nine hundred and ninety-two participants who had not completed the lifestyle and/or diet questionnaires were excluded from the present study (Figure 1).

All procedures were performed according to the ethical standards of the institutional research committee of the National Institute of Gastroenterology, IRCCS “S. De Bellis” Research Hospital, after the ethics committee had approved the MICOL study (DDG-CE-589/2004 18 November 2004).

The study was conducted following the 1964 Helsinki Declaration and its later amendments, and written informed consent was obtained from each participant.

### 2.1. Data Collection 

The participants were interviewed to collect information on their sociodemographic characteristics, health status, personal history, and lifestyle factors, including their tobacco use (never and current), educational level (none, primary school, secondary school, high school, graduate), job (pensioners and unemployed, managers and professionals, crafts, agricultural and sales workers, housewives, and elementary occupations), and marital status (single, married/coupled, separated/divorced, and widow/er).

Weight was measured with the subject in their underwear, standing on an electronic balance, from SECA^®^, and approximated to the nearest 0.1 kg. Height was measured with a wall-mounted stadiometer, from SECA^®^, approximated to 1 cm. Blood pressure (BP) measurement was performed following international guidelines. The average of 3 BP measurements was calculated. 

Habitual food intake and LTPA were estimated by the self-administration of a validated Food Frequency Questionnaire (FFQ) and checked with the support of qualified personnel. The FFQ was built to capture the dietary habits of the Apulian population, as well as their physical activity during daily living [12].

Fasting venous blood samples were drawn, and the serum was separated into two aliquots. One aliquot was immediately stored at −80 °C. The second aliquot was used to test biochemical serum markers by standard laboratory techniques in our Clinical Pathology Laboratory.

### 2.2. Outcome Assessment

The outcome of this study was the all-cause mortality of subjects enrolled in the MICOL cohort.

Information on the vital status of the participants was obtained both from the municipality of Castellana Grotte and the municipalities of current residence in the case of subjects who had migrated during the follow-up. This information was linked electronically to the database. 

### 2.3. Exposure Assessment

#### 2.3.1. Definition of MASLD

As published in previous studies [8,13], the definition of MASLD was based on the presence of hepatic steatosis plus at least 1 of the following 5 conditions:

(1) BMI > 25 kg/m^2^ or waist circumference >94 cm in men and >80 in women; (2) fasting serum glucose ≥ 100 mg/dL (≥5.6 mmol/L), 2 h post-load glucose level ≥ 140 mg/dL (≥7.8 mmol/L), HbA1c ≥ 5.7%, or on specific drug treatment; (3) blood pressure ≥ 130/85 mmHg or on specific drug treatment; (4) plasma triglycerides ≥ 150 mg/dL (≥1.70 mmol/L) or on specific drug treatment; (5) plasma HDL cholesterol < 40 mg/dL (<1.0 mmol/L) for men and <50 mg/dL (<1.3 mmol/L) for women or on specific drug treatment.

Furthermore, the definition of MASLD continued to limit alcohol intake (as previously restricted for NAFLD) in the context of steatosis: average daily intake 20–50 g in women and 30–60 g in men [3].

Finally, the coexistence of other forms of liver disease with MASLD, e.g., MASLD + HCV, was excluded so as not to alter the natural history of the disease.

Hepatic steatosis for individuals in the MICOL study was assessed by liver ultrasound (LUS) (Esaote MyLab70 XVG device and Convex 5-MHz probe).

#### 2.3.2. Physical Activity Measurement

PA was assessed by a questionnaire probing the frequency and intensity of activity performed during a typical week, including walking, cycling, exercising, and all types of leisure hobbies. The intensity of each activity was measured with the corresponding metabolic equivalent of the task (MET), following the Physical Activity Compendium [14]. 

LTPA includes recreational and sports activities such as cycling, gym use, and hobbies (bricolage, painting, sewing, etc.), considering the number of days, hours, and minutes spent on each activity during the week.

The subjects, based on the total METs of the LTPA, were assigned to 2 groups—absent and low LTPA (<700 METs) and moderate and high LTPA (>701 METs)—referring to the International Physical Activity Questionnaire (IPAQ) categories [15].

### 2.4. Statistical Analysis

For analytical purposes, we combined the association between MASLD (absence or presence) and leisure-time activity levels (high and moderate versus low and absent).

Data are presented as the mean (±SD) or median (±IQR) for continuous data or a frequency (%) for categorical data.

The observation time was the time from enrolment to death, moving elsewhere, or the end of the study (31 December 2023), whichever occurred first.

Since age is the most important risk factor for death, we chose age at death as the time scale.

We considered 90 years of age as the maximum observation age at death to avoid problems related to comorbidities in the over 90s, worse coding quality for deaths occurring in very old age, and the small number of deaths beyond this age.

Schoenfeld residuals were calculated to test the proportional hazards assumption (Figure S1).

We used flexible parametric survival models [16] with 3 degrees of freedom and with age at death as the underlying time metric to estimate the hazard ratios (HR) and 95% confidence intervals (CI) for the association between MASLD combined with LTPA and all-cause mortality. 

A selection procedure (LASSO Cox) was adopted to reduce the number of candidate predictors, select those most useful to build the survival model, and verify that MASLD and LTPA were risk factors (Figure S2). 

The flexible parametric survival models were adjusted for the following covariates: gender (M vs. F), age (<50 years vs. ≥50 years), smoking habit, job, education, rMED, triglycerides, daily consumption of wine (mL), daily kilocalories, and SBP. 

We constructed six models. Model 1 included MASLD (absence versus presence) and the LTPA level (high and moderate vs. low and absent), adjusted by the above-mentioned covariates.

Models 2 (females) and 3 (males) were the same as model 1 but stratified by gender.

In model 4, we built a new variable that resulted from the combination of MASLD (absence or presence) and the LTPA levels and was adjusted by the same covariates.

Models 5 (females) and 6 (males) were the same as model 4 but stratified by gender.

In models 4, 5, and 6, we chose no MASLD and a high or moderate LTPA level as the reference group. Using post-estimation tools, we predicted the overall and gender-specific survival, which we used to display the survival functions graphically.

Taking the counterfactual approach [17], we estimated the total effect (TE), natural direct effect (NDE), natural indirect effect (NIE), and proportion mediated (PM) by gender as mediator variables between MASLD/LTPA and the risk of death. Firstly, we predicted survival among men (s0m0) and women (s1m1), and then we predicted female survival if they had the male MASLD/LTPA distribution (s1m0) and male survival if they had the female MASLD/LTPA distribution (s0m1). Subsequently, we estimated the TE (s0m0-s1m1), NDE (s0m0–s1m0), NIE (s1m0–s1m1), and PM (NIE/TE) of gender [18].

All statistical analyses were performed using the Stata statistical software, version 18.0 (StataCorp, 4905 Lakeway Drive, College Station, TX 77845, USA). In particular, the stpm3 official Stata command and its post-estimation commands, such as predict, were used.

## 3. Results

### 3.1. Cohort Characteristics

The main characteristics of the 1826 participants, classified according to the four combinations of MASLD (absence and presence) and LTPA levels (high and moderate vs. low and absent), are shown in Table 1. Overall, 38.7 percent of the whole sample had MASLD, most of which (67.6%) were male.

During the follow-up [17.92 (17.05–18.22)], 374 (20.5%) participants died. The study base produced a total of 11,264.612 person-years (pyrs), with an incidence rate of 37.2 for 1000 pyrs.

The mean age of the 1452 participants still alive at the end of the follow-up was 65.22 years (±11.90), while the average age at death was 80.31 years (±11.01), being 82.71 (±9.57) for women and 78.39 (±11.72) years old for men.

Details on the characteristics of the participants are shown in Table S1.

The subjects who answered the questionnaire fully and were therefore included in the project were mostly women. They were younger than the excluded subjects and had a higher level of education. There was no statistically significant difference between the two groups in the presence of MASLD.

### 3.2. Effect of MASLD and LTPA Level Categories for Whole Sample and Stratified by Gender

Table 2 shows the results of the flexible parametric survival models for the whole sample and stratified by gender.

After adjustment, statistically significantly higher HRs were evident among participants with MASLD and among those who exhibited low or absent LTPA levels in the whole sample (MASLD: HR 1.30, 95% CI 1.00; 1.67; low or absent LTPA level: HR 1.45, 95% CI 1.10; 1.91) and among females (low or absent LTPA level: HR 1.64 95% CI 1.07; 2.50).

### 3.3. Effect of Combining MASLD and LTPA Level Categories for Whole Sample and Stratified by Gender

The results of the flexible parametric survival models for the combination of MASLD and the LTPA level categories for the whole sample and stratified by gender are shown in Table 3.

There were statistically significant HRs for the combination of MASLD/LTPA low or absent for all-cause mortality in the whole sample (HR 2.23 95% CI 1.49; 3.34), females (HR 3.10 95% CI 1.69; 5.68), and males (HR 1.84 95% CI 1.07; 3.16). No statistical significance was found for the other categories.

**Figure 2:**
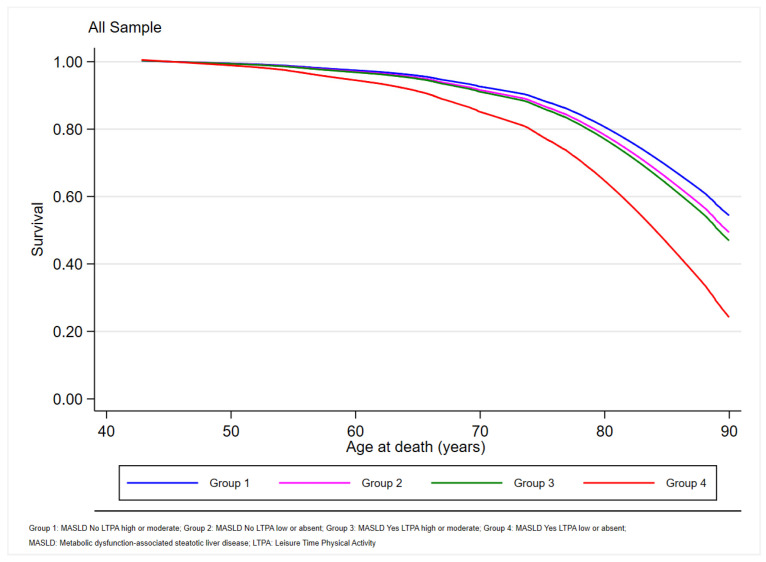
Predicted survival for the combination of MASLD and LTPA level categories regarding all-cause mortality.

The graph in Figure 2 displays the predicted survival for the combination of MASLD and the LTPA level categories regarding all-cause mortality.

The survival functions were statistically different for the different categories of MASLD/LTPA (*p*-value < 0.001) and for the trends (*p*-value < 0.001) of the survival functions. When the analysis excluded the combination of MASLD/LTPA low or absent, there was no longer a statistically significant difference in the survival functions (*p*-value = 0.17).

**Figure 3:**
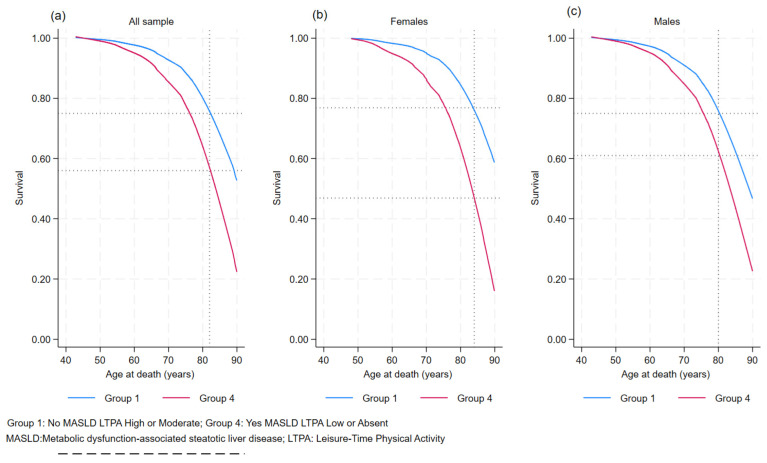
Survivor function for whole sample (**a**), females (**b**), and males (**c**) regarding all-cause mortality with MASLD/LTPA exposure. MICOL study, Castellana Grotte (BA), 2005–2006–2023.

The graph in Figure 3 displays the survival functions for the two extreme exposure categories of MASLD/LTPA for the whole sample and stratified by gender, where an increase in the survival probability from 50 years old at death (first event) to 90 years old at death was observed. The graph also displays the life expectancy for the general population, females, and males in the Apulian region.

At age 82, the survival probability for those who had MASLD/LTPA low or absent and MASLD/LTPA high or moderate was 56% and 75%, respectively. In females, at age 84, the survival probability for those with MASLD/LTPA low or absent and MASLD/LTPA high or moderate was 47% and 77%, respectively. At age 80, the survival probability for males with MASLD/LTPA low or absent and MASLD/LTPA high or moderate was 61% and 75%, respectively.

**Figure 4:**
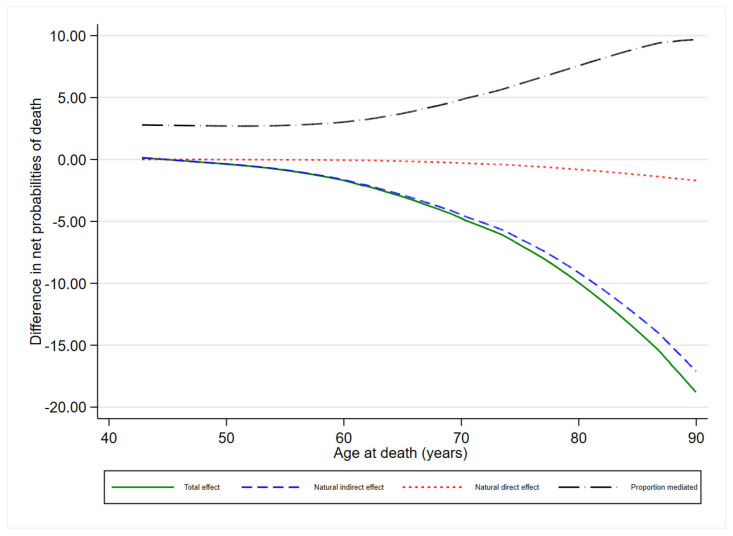
MASLD/LTPA total, natural direct, and natural indirect effect and proportion mediated by gender on the risk of all-cause mortality. MICOL study, Castellana Grotte (BA), 2005–2006–2023.

Figure 4 displays the TE, NDE, and NIE of MASLD/LTPA and the PM of gender regarding the mortality risk. 

There was an increasing TE for MASLD/LTPA regarding the risk of mortality from 50 years old (−1% survival probability) to 90 years old at death (−18% survival probability). The total effect was almost entirely due to the NDE. A weak NIE is evident from 50 years old (near 0) to 90 years old at death (about −2% survival probability). As a consequence, the PM by gender increases from 50 to 90 years old at death.

## 4. Discussion

Obesity and adiposity are the main factors behind the development of MASLD, a major chronic liver disease worldwide [19]. It is closely linked to the development of insulin resistance and type 2 diabetes mellitus (T2DM), hypertension, and dyslipidemia [20]. It is, therefore, a disease directly related to an unhealthy lifestyle (unhealthy diet and sedentary lifestyle). 

This study showed that subjects with MASLD who practiced low LTPA had a significantly higher risk of death compared to those who did not have MASLD and practiced high LTPA. Furthermore, subjects with MASLD who practiced low LTPA were approximately 19% less likely to survive to the age of 82.

As regards gender, both men and women with MASLD and low LTPA show a significant risk of death, but this is actually higher in women. 

MASLD is a very dynamic disease with slow progression, whereby the transition from steatosis to liver fibrosis occurs silently [19]. Recent studies have analyzed NHANES III data comparing the risk of death from all causes in groups of individuals with NAFLD, MAFLD, and MASLD [21,22]. They found that MASLD and MAFLD were associated with a higher risk of death than NAFLD. However, MASLD, compared to MAFLD, affects more older subjects (>65 years), probably due to the presence of cardiometabolic risk factors [23].

As with NAFLD, men seem to be more frequently affected by MASLD than women, but this unequal distribution is related to the age variable [24]. In fact, the condition primarily affects men when they are young, but, as they age, it increasingly affects women, probably due to the loss of estrogen as a protective factor that occurs from menopause onwards [25]. Our results support this hypothesis as we have seen that women with MASLD have a statistically significantly higher mortality risk than men.

Disease regression can occur with weight loss [26]. Therefore, lifestyle modification, i.e., a healthy diet and PA, should be implemented to avoid worsening MASLD and to reduce the metabolic risk factors leading to cardiovascular disease [9].

Even when practiced alone, PA in individuals with MASLD is known to reduce hepatic steatosis and liver enzyme levels; it also impacts the control of glucose and lipid metabolism [27]. Endurance PA slows down the progression of MASLD; it lowers energy consumption, aids in the preservation of muscle mass, and increases strength [28]. In addition, muscle mass stimulates the release of interleukin-6 (IL-6), which acts mainly in glucose and lipid metabolism [29]. Aerobic activity, on the other hand, reduces several metabolic parameters, acting positively in subjects with MASLD [30]. It can contribute to weight reduction, especially regarding the percentage of fat mass [31]. 

However, not all types of PA have the same beneficial effects on the disease. In fact, following the guidelines proposed by the NHANES studies, non-occupational PA appears to have an important impact on risk reduction in NAFLD [8]. In contrast, occupational activity does not produce the same direct effects as LTPA. Byambasuck showed that the potential effects of PA on NAFLD depend on the type of daily activity performed [32].

In individuals with MASLD, PA can reduce all-cause mortality, having an important impact on survival [9]. Furthermore, our study showed that low levels of LTPA promote a significant risk of death compared to moderate and high levels. Regular PA, especially during leisure time, has a positive and protective impact on all causes of death [33]. Increased amounts and intensity of PA can improve fitness and help to reduce the risk of all-cause mortality [33].

Machado and Li related LTPA to MASLD, verifying that, in the total PA practiced, LTPA exhibits a difference compared to MASLD and that recreational and leisure activities are associated with weight loss and protective effects against hepatic steatosis [8,19]. Our results confirm that individuals with MASLD who practice low LTPA have a significant risk of death compared to those with moderate and high LTPA levels.

However, when considered by gender, both men and women with MASLD with low LTPA levels have a significant risk of death, although this is actually significantly higher in women.

LTPA is statistically negatively correlated with CAP in women and younger groups, probably due to hormonal differences [34]. Gender hormones play an important role in the regulation of glucose and lipid metabolism in the liver; the decline of these hormones in the postmenopausal period contributes to the altered distribution of visceral fat and favors the development of hepatic steatosis [35]. Ballestri et al. [36] demonstrated that NAFLD is a condition influenced by gender differences, and exploring this correlation could explain how PA impacts and reduces the severity of NAFLD. As shown by Lollgen, women generally tend to be less active than men [33]. This could support our results showing a significantly higher risk of death in women with MASLD who have low LTPA. Furthermore, for women, less activity at a young age poses a greater risk for later life, as the hormonal protection against cardiovascular disease present in the menopausal period is lost [37]. Therefore, women should be more active in later life so as to benefit from the lower risk of death of men.

Further research is needed to better understand the role of gender differences in MASLD and how these may influence the risk of death in relation to PA levels.

This study has various strengths and limitations. There are few studies on MASLD and PA levels in the literature, so this may be a starting point to investigate the relationship between mortality in individuals with MASLD and LTPA. Being a population-based study, it is reasonable to conclude that the distribution of the main exposure (MASLD) reflects the whole population. LTPA may differ between included and non-included participants as the age–gender distribution may be different. However, if there is selection bias, it could be self-selection bias, a specific case in which the biasing forces affect a subset of the participants [38]. Although the sample size is sufficiently large and our estimates are reliable, it must be remembered that the data collected through the questionnaire were self-reported, and therefore a “socially desirable” response bias may exist. Finally, as the LTPA exposure is probably a time-dependent exposure, the counterfactual approach applied to estimate the TE, NDE, NIE, and PM may be slightly biased.

## 5. Conclusions

The presence of MASLD, especially in women, increases the risk of death from all causes. In individuals with this disease, LTPA plays a key role; in fact, practicing low levels of leisure-time activity increases the risk of death compared to moderate and high LTPA. The risk is higher in women than in men because of gender hormone differences, which lead to a worse prognosis in the postmenopausal period. Therefore, it is essential, especially in old age, for women to engage in regular physical activity in their free time to benefit from its protective effect against disease-related risks.

It is, therefore, essential to inform those with MASLD of the benefits of PA, not only of a structured type but also practiced in everyday settings during leisure time, to reduce the risk of death.

## Figures and Tables

**Table 1:** Characteristics of participants by MASLD and level of leisure activity (LTPA). MICOL/PANEL study. Castellana Grotte (BA), Italy, 2005–2023.

		MASLD and Leisure Activity Level			
	Whole Sample ^d^	Group 1	Group 2	Group 3	Group 4
N (%)	1826	909	210	551	156
Enrolment age (years) ^a^	51.91 (14.76)	50.45 (15.30)	53.50 (16.23)	53.43 (13.44)	52.91 (13.16)
Gender ^b^					
Males	1026 (56.2)	449 (43.8)	99 (9.6)	371 (36.2)	107 (10.4)
Females	800 (43.8)	460 (57.5)	111 (13.9)	180 (22.5)	49 (6.1)
SBP (mmHg) ^a^	122.13 (19.92)	118.43 (20.00)	122.14 (20.91)	126.27 (17.94)	128.99 (20.21)
DBP (mmHg) ^a^	74.67 (10.14)	72.71 (9.73)	73.89 (10.26)	77.42 (9.86)	77.32 (10.55)
Weight (kg) ^a^	75.75 (15.53)	70.21 (12.44)	70.61 (14.56)	84.03 (14.94)	85.66 (17.18)
BMI (kg/m^2^) ^a^	28.68 (5.27)	26.63 (4.16)	27.29 (4.73)	31.54 (4.96)	32.39 (6.19)
Waist circumference (cm) ^a^	92.56 (13.41)	86.94 (11.48)	88.20 (12.28)	100.65 (11.20)	102.33 (12.49)
Hip circumference (cm) ^a^	103.61 (10.46)	100.28 (8.82)	101.49 (9.26)	108.25 (10.43)	109.26 (12.23)
WHR ^a^	0.89 (0.08)	0.87 (0.08)	0.87 (0.08)	0.93 (0.07)	0.94 (0.07)
Kilocalories ^a^	2171.26 (698.05)	2180.6 (688.7)	2089.5 (702.0)	2178.3 (693.1)	2201.8 (760.9)
Alcohol consumption (g/die) ^a^	11.87 (14.74)	10.69 (13.86)	9.98 (13.84)	13.98 (15.92)	13.82 (15.60)
Wine consumption (ml/die) ^a^	106.06 (136.67)	96.36 (128.44)	88.51 (128.66)	124.69 (147.59)	120.43 (145.78)
TG (mg/dL) ^a^	128.37 (96.38)	10.69 (13.86)	9.98 (13.84)	13.98 (15.92)	13.82 (15.60)
TC (mg/dL) ^a^	199.60 (38.00)	96.36 (128.44)	88.51 (128.66)	124.69 (147.59)	120.43 (145.78)
HDL-C (mg/dL) ^a^	50.79 (13.81)	10.69 (13.86)	9.98 (13.84)	13.98 (15.92)	13.82 (15.60)
LDL-C (mg/dL) ^a^	123.41 (32.83)	96.36 (128.44)	88.51 (128.66)	124.69 (147.59)	120.43 (145.78)
Glucose (mg/dL) ^a^	108.66 (25.10)	10.69 (13.86)	9.98 (13.84)	13.98 (15.92)	13.82 (15.60)
ALT (U/L) ^a^	17.24 (12.86)	96.36 (128.44)	88.51 (128.66)	124.69 (147.59)	120.43 (145.78)
Smoking habit ^b^					
Never	1504 (82.4)	752 (50.0)	182 (12.1)	454 (30.2)	116 (7.7)
Current	322 (17.6)	157 (48.8)	28 (8.7)	97 (30.1)	40 (12.4)
rMED ^c^	8 (7–10)	8 (7–10)	8 (7–10)	8 (7–10)	8 (6–9)
Age at death (years) ^c^	65.83 (56.86–79.05)	63.41 (55.82–78.90)	65.56 (56.34–82.35)	69.81 (60.13–80.07)	70.35 (60.17–76.61)
Observation time (years) ^c^	17.92 (17.05–18.22)	17.91 (17.05–18.13)	17.91 (17.02–18.20)	17.92 (17.05–18.33)	17.93 (17.03–18.42)
Status ^b^					
Alive	1452 (79.5)	739 (50.9)	157 (10.8)	438 (30.2)	118 (8.1)
Dead	374 (20.5)	170 (45.5)	53 (14.2)	113 (30.2)	38 (10.2)
Job ^b^					
Unemployed and pensioner	385 (21.2)	182 (47.3)	45 (11.7)	133 (34.5)	25 (6.5)
Manager and professional	118 (6.5)	58 (49.2)	21 (17.8)	28 (23.7)	11 (9.3)
Crafts, agricultural, and sales worker	627 (34.6)	306 (48.8)	68 (10.8)	194 (30.9)	59 (9.4)
Housewife	220 (12.1)	134 (60.9)	18 (8.2)	55 (25.0)	13 (5.9)
Elementary occupation	462 (25.5)	222 (48.1)	57 (12.3)	137 (29.7)	46 (10.0)
Education ^b^					
Primary school	622 (34.1)	281 (45.2)	78 (12.5)	209 (33.6)	54 (8.7)
Secondary school	568 (31.1)	290 (51.1)	63 (11.1)	170 (29.9)	45 (7.9)
High school	517 (28.3)	270 (52.2)	58 (11.2)	141 (27.3)	48 (9.3)
Graduate	119 (6.5)	68 (57.1)	11 (9.2)	31 (26.1)	9 (7.6)
Marital status ^b^					
Single	224 (12.3)	131 (58.5)	34 (15.2)	43 (19.2)	16 (7.1)
Married or cohabiting	1.403 (76.8)	672 (47.9)	155 (11.0)	456 (32.5)	120 (8.6)
Separated or divorced	58 (3.2)	35 (60.3)	4 (6.9)	17 (29.3)	2 (3.4)
Widow/er	141 (7.7)	71 (50.4)	17 (12.1)	35 (24.8)	18 (12.8)
Hypertension ^b^					
No	1280 (70.1)	691 (54.0)	148 (11.6)	346 (27.0)	95 (7.4)
Yes	546 (29.9)	218 (39.9)	62 (11.4)	205 (37.5)	61 (11.2)
Dyslipidemia ^b^					
No	1360 (74.5)	732 (53.8)	170 (12.5)	357 (26.2)	101 (7.4)
Yes	466 (25.5)	177 (38.0)	40 (8.6)	194 (41.6)	55 (11.8)
Diabetes ^b^					
No	1664 (91.1)	856 (51.4)	191 (11.5)	478 (28.7)	139 (8.4)
Yes	162 (8.9)	53 (32.7)	19 (11.7)	73 (45.1)	17 (10.5)

Group 1: MASLD no, LTPA high and moderate; Group 2: MASLD no, LTPA low and absent; Group 3: MASLD yes, LTPA high and moderate; Group 4: MASLD yes, LTPA low and absent. MASLD: metabolic-dysfunction-associated steatotic liver disease; LTPA: leisure-time physical activity; SBP: systolic blood pressure; DBP: diastolic blood pressure; BMI: body mass index; TG: triglycerides; TC: total cholesterol; HDL-C: high-density lipoprotein cholesterol; LDL-C: low-density lipoprotein cholesterol; ALT: alanine amino transferase; rMED: Mediterranean relative scoring system. ^a^ Mean (±SD); ^b^ number (percentage); ^c^ median (IQR); ^d^ percentages calculated for the column; otherwise, percentages calculated for the row.

**Table 2:** Flexible parametric survival models: HRs and 95% confidence intervals for the effect of MASLD and the LTPA level categories on all-cause mortality for the whole sample and stratified by gender. MICOL study, Castellana Grotte (BA), 2005–2006–2023.

	Model 1	Model 2	Model 3
	Whole Sample	Female	Male
	HR (95% CI)	HR (95% CI)	HR (95% CI)
MASLD			
No	1.00	1.00	1.00
Yes	1.30 * (1.00; 1.67)	1.27 (0.83; 1.95)	1.30 (0.94; 1.81)
LTPA			
High or Moderate	1.00	1.00	1.00
Low or Absent	1.45 * (1.10; 1.91)	1.64 * (1.07; 2.50)	1.34 (0.92; 1.94)

* *p*-value < 0.05. Model 1 adjusted for gender (M vs. F), age at enrollment (<50 years vs. ≥50 years), smoking habit, job, education, rMED, triglycerides, wine consumption (ml/die), daily kilocalories, systolic blood pressure. Models 2 and 3 (female and male) adjusted in the same way as model 1, except for gender. HR: hazard ratio; MASLD: metabolic-dysfunction-associated steatotic liver disease; LTPA: leisure-time physical activity; rMED: Mediterranean relative scoring system.

**Table 3:** Flexible parametric survival models: HRs and 95% confidence intervals for the effect of the combination of MASLD and the LTPA level categories on all-cause mortality for the whole sample and stratified by gender. MICOL study, Castellana Grotte (BA), 2005–2006–2023.

	Model 4	Model 5	Model 6
	Whole Sample	Females	Males
	HR (95% CI)	HR (95% CI)	HR (95% CI)
MASLD#LTPA			
Group 1	1.00	1.00	1.00
Group 2	1.18 (0.83; 1.68)	1.16 (0.69; 1.93)	1.18 (0.72; 1.95)
Group 3	1.10 (0.83; 1.46)	0.92 (0.57; 1.49)	1.17 (0.82; 1.67)
Group 4	2.23 ** (1.49; 3.34)	3.10 ** (1.69; 5.68)	1.84 * (1.07; 3.16)

Group 1 reference category. * *p*-value < 0.05 ** *p*-value < 0.001. Model 4 adjusted for gender (M vs. F), age at enrolment (<50 years vs. ≥50 years), smoking habit, job, education, rMED, triglycerides, wine consumption (ml/die), daily kilocalories, systolic blood pressure. Models 5 and 6 (females and males) adjusted similarly to model 4, except for gender. Group 1: no MASLD and LTPA high or moderate; Group 2: no MASLD and LTPA low or absent; Group 3: MASLD and LTPA high or moderate; Group 4: MASLD and LTPA low or absent. HR: hazard ratio; MASLD: metabolic-dysfunction-associated steatotic liver disease; LTPA: leisure-time physical activity; rMED: Mediterranean relative scoring system.

## Data Availability

The data are available by contacting the corresponding author at the email address antonella.bianco@irccsdebellis.it.

## Supplementary Materials

The following supporting information can be downloaded at: https://www.mdpi.com/article/10.3390/jcm13133772/s1, Figure S1: Test of proportional- hazards assumption; Figure S2: Selection procedure for LASSO Cox. Table S1: Participant characteristics based on the completion of the LTPA questionnaire, MICOL/PANEL, Castellana Grotte (BA), Italy, 2005–2023.
