# Supplementary material for: Leisure-Time Physical Activity in Subjects with Metabolic-Dysfunction-Associated Steatotic Liver Disease: An All-Cause Mortality Study
Source: J Clin Med. 2024 Jun 27;13(13):3772. doi: 10.3390/jcm13133772 (PMC11242783; doi:10.3390/jcm13133772)
Supplement: Supplementary file 1 [file jcm-13-03772-s001.zip › jcm-3066934-supplementary.pdf]

Table S1. Participant characteristics are based on the completion of the LTPA questionnaire. MICOL/PANEL. Castellana Grotte (BA). Italy. 2005-2023

	Questionnaire LTPA		
	No	Yes	*p-value*[¥]
N	777	1972	
Enrollment Age (years)[a]	61,24 (13,70)	52,19 (14,68)	<0.001
Gender [b]			
Male	366 (47.1)	825 (41.8)	0.012
Female	411 (52.9)	1,147 (58.2)	
MASLD [b]			
No	459 (59.1)	1,157 (58.7)	0.85
Yes	318 (40.9)	815 (41.3)	
SBP (mmHg) [a]	126,09 (19,70)	122,66 (19,77)	<0.001
DBP (mmHg) [a]	74,46 (10,32)	74,92 (10,05)	0.28
Weight (kg) [a]	72,82 (14,52)	76,08 (15,46)	<0.001
BMI (kg/m^2) [a]	28,65 (5,13)	28,75 (5,22)	0.65
Waist circumference (cm) [a]	92,93 (13,28)	93,04 (13,36)	0.86
Kilocalories [a]	2197,8 (712,5)	2201,26 (713,9)	0.91
Alcohol consumption (g/die) [a]	16,29 (21,69)	15,02 (20,51)	0.15
Wine consumption (ml/die) [a]	152,97 (202,64)	135,69 (191,80)	0.036
TG (mg/dL) [a]	128,82 (81,84)	129,15 (95,85)	0.93
TC (mg/dL) [a]	198,96 (39,08)	199,72 (38,42)	0.65
HDL-C (mg/dL) [a]	52,35 (14,44)	50,71 (13,76)	0.006
LDL-C (mg/dL) [a]	120,61 (34,30)	123,32 (32,84)	0.056
Glucose (mg/dL) [a]	109,37 (30,88)	109,11 (25,27)	0.82
ALT (U/L) [a]	17,40 (18,39)	17,84 (13,98)	0.50
Smoking habit [b]			
Never	657 (84.6)	1,611 (81.7)	0.075
Current	120 (15.4)	361 (18.3)	
rMED [c]	8,00 (7,00-10,00)	8,00 (7,00-9,00)	0.09
Job [b]			
Managers & Professionals	14 (1.8)	106 (5.4)	<0.001
Craft, Agricultural and Sales Workers	142 (18.3)	545 (27.6)	
Elementary Occupations	187 (24.1)	500 (25.4)	
Housewife	100 (12.9)	212 (10.8)	
Pensioneers	322 (41.4)	579 (29.4)	
Jobless	12 (1.5)	30 (1.5)	
Education [b]			
Primary School	304 (39.3)	617 (31.3)	<0.001
Secondary School	244 (31.5)	606 (30.7)	
High School	156 (20.2)	503 (25.5)	
Graduated	70 (9.0)	245 (12.4)	
Marital Status [b]			

Single	67 (8.6)	232 (11.8)	0.005
Married or Cohabiting	603 (77.6)	1,522 (77.2)	
Separated or Divorced	20 (2.6)	63 (3.2)	
Widower	87 (11.2)	155 (7.9)	
Hypertension [b]			
No	450 (57.9)	1,370 (69.5)	<0.001
Yes	327 (42.1)	602 (30.5)	
Dyslipidemia [b]			
No	565 (72.7)	1,471 (74.6)	0.31
Yes	212 (27.3)	501 (25.4)	
Diabetes [b]			
No	681 (87.6)	1,794 (91.0)	0.009
Yes	96 (12.4)	178 (9.0)	

[¥]Wilcoxon rank-sum for continuous variables and Pearson's Chi-square for categorical. MASLD: Metabolic dysfunction-associated steatotic liver disease; LTPA: Leisure Time Physical Activity; SBP: Systolic Blood Pressure; DBP: Diastolic Blood Pressure; BMI: Body Mass Index; TG: Triglycerides TC: Total Cholesterol; HDL-C: High-Density Lipoprotein Cholesterol; LDL-C: Low-Density Lipoprotein Cholesterol; ALT: Alanine Aminotransferase; rMED: Mediterranean relative scoring system.

[a]Mean ± (SD), [b]Number (Percentage), [c]Median (IQR). Percentages calculated for the row.

Table S2. Characteristics of Females by MASLD and level leisure activities (LTPA). MICOL/PANEL Study. Castellana Grotte (BA). Italy. 2005-2023.

	MASLD and Leisure Activities Level			
	Group 1	Group 2	Group 3	Group 4
N (%)	460 (57.50)	111 (13.88)	180 (22.50)	49 (6.12)
Enrolment Age (years)[a]	51.34 (15.21)	55.81 (16.03)	58.39 (12.43)	56.80 (13.95)
SBP (mmHg) [a]	116.65 (20.95)	122.00 (23.06)	128.33 (19.70)	132.24 (20.94)
DBP (mmHg) [a]	71.79 (9.82)	72.86 (10.45)	76.12 (10.20)	78.78 (11.76)
Weight (kg) [a]	64.55 (11.16)	64.56 (12.49)	77.73 (15.16)	83.22 (20.79)
BMI (kg/m^2) [a]	26.67 (4.84)	27.29 (5.43)	33.29 (5.62)	35.39 (7.81)
Waist circumference (cm) [a]	82.11 (11.41)	83.25 (11.14)	97.15 (11.67)	100.95 (14.57)
Hip circumference (cm) [a]	100.45 (10.03)	101.24 (10.12)	111.68 (11.52)	115.86 (14.66)
WHR [a]	0.82 (0.06)	0.82 (0.05)	0.87 (0.05)	0.87 (0.05)
Kilocalories [a]	1986.03 (622.97)	1974.19 (653.67)	1987.55 (720.05)	1993.41 (790.49)
Alcohol consumption (g/die) [a]	5.52 (9.29)	5.85 (10.02)	5.96 (10.77)	7.91 (13.36)
Wine consumption (ml/die) [a]	53.40 (92.14)	57.53 (100.18)	56.41 (102.51)	74.85 (123.16)
TG (mg/dL) [a]	92.10 (49.49)	94.19 (52.58)	154.49 (97.34)	157.67 (129.90)
TC (mg/dL) [a]	196.43 (35.72)	194.59 (32.74)	209.56 (40.85)	200.04 (46.07)
HDL-C (mg/dL) [a]	59.02 (15.61)	57.01 (13.95)	50.67 (11.78)	48.82 (10.66)
LDL-C (mg/dL) [a]	119.03 (31.41)	117.86 (30.32)	127.77 (35.44)	119.41 (38.29)
Glucose (mg/dL) [a]	100.91 (18.50)	106.80 (28.57)	116.82 (31.42)	115.59 (29.21)
ALT (U/L) [a]	13.23 (10.78)	13.86 (10.50)	15.99 (9.96)	17.45 (8.99)
Smoking habit [b]				

Never	399 (56.4)	103 (14.6%)	162 (22.9%)	43 (6.1%)
Current	61 (65.6)	8 (8.6%)	18 (19.4%)	6 (6.5%)
rMED [c]	8.00 (7.00-10.00)	8.00 (7.00-10.00)	8.00 (7.00-10.00)	8.00 (7.00-9.00)
Age at Death (years) [c]	64.20 (56.48-80.00)	73.23 (59.35-83.56)	76.23 (66.28-84.08)	70.95 (64.90-79.31)
Observation Time (years) [c]	17.94 (17.09-18.20)	17.91 (17.02-18.29)	18.09 (17.09-18.47)	17.89 (10.83-18.20)
Status [b]				
Alive	381 (60.1)	82 (12.9%)	140 (22.1%)	31 (4.9%)
Dead	79 (47.6)	29 (17.5%)	40 (24.1%)	18 (10.8%)
Job [b]				
Unemployed & Pensioner	79 (48.5)	26 (16.0%)	49 (30.1%)	9 (5.5%)
Manager & Professional	18 (66.7)	7 (25.9%)	1 (3.7%)	1 (3.7%)
Crafts. Agricultural and Sales Worker	126 (64.3)	32 (16.3%)	29 (14.8%)	9 (4.6%)
Housewife	133 (60.7)	18 (8.2%)	55 (25.1%)	13 (5.9%)
Elementary Occupation	98 (53.8)	27 (14.8%)	42 (23.1%)	15 (8.2%)
Education [b]				
Primary School	168 (48.1)	52 (14.9%)	101 (28.9%)	28 (8.0%)
Secondary School	135 (65.2)	20 (9.7%)	42 (20.3%)	10 (4.8%)
High School	122 (62.2)	36 (18.4%)	29 (14.8%)	9 (4.6%)
Graduate	35 (72.9)	3 (6.2%)	8 (16.7%)	2 (4.2%)
Marital Status [b]				
Single	56 (66.7)	14 (16.7%)	8 (9.5%)	6 (7.1%)
Married or Cohabiting	320 (56.6)	77 (13.6%)	139 (24.6%)	29 (5.1%)
Separated or Divorced	22 (66.7)	3 (9.1%)	6 (18.2%)	2 (6.1%)

Widow/er	62 (52.5)	17 (14.4%)	27 (22.9%)	12 (10.2%)
Hypertension [b]				
No	342 (63.9)	74 (13.8%)	93 (17.4%)	26 (4.9%)
Yes	118 (44.5)	37 (14.0%)	87 (32.8%)	23 (8.7%)
Dyslipidemia [b]				
No	374 (61.2)	88 (14.4%)	115 (18.8%)	34 (5.6%)
Yes	86 (45.5)	23 (12.2%)	65 (34.4%)	15 (7.9%)
Diabetes [b]				
No	438 (59.6)	102 (13.9%)	150 (20.4%)	45 (6.1%)
Yes	22 (33.8)	9 (13.8%)	30 (46.2%)	4 (6.2%)

Group 1: MASLD No LTPA high and moderate; Group 2: MASLD No LTPA low and absent; Group 3: MASLD Yes LTPA high and moderate; Group 4: MASLD Yes LTPA low and absent; MASLD: Metabolic dysfunction-associated steatotic liver disease; LTPA: Leisure Time Physical Activity; SBP: Systolic Blood Pressure; DBP: Diastolic Blood Pressure; BMI: Body Mass Index; TG: Triglycerides TC: Total Cholesterol; HDL-C: High-Density Lipoprotein Cholesterol; LDL-C: Low-Density Lipoprotein Cholesterol; ALT: Alanine Amino transferase, rMED: Mediterranean relative scoring system [a] Mean (±SD); [b] Number (Percentage), [c] Median (IQR). [d]Percentages calculated for the column. Otherwise percentages calculated for the row.

Table S3. Characteristics of Males by MASLD and level leisure activities (LTPA). MICOL/PANEL Study. Castellana Grotte (BA). Italy. 2005-2023.

	MASLD and Leisure Activities Level			
	Group 1	Group 2	Group 3	Group 4
N (%)	449 (43.76)	99 (9.65)	371 (36.16)	107 (10.43)
Enrolment Age (years)[a]	49.54 (15.34)	50.90 (16.13)	51.03 (13.27)	51.13 (12.44)
SBP (mmHg) [a]	120.23 (18.83)	122.30 (18.29)	125.26 (16.94)	127.50 (19.79)
DBP (mmHg) [a]	73.65 (9.55)	75.06 (9.96)	78.06 (9.65)	76.65 (9.94)
Weight (kg) [a]	76.01 (10.92)	77.40 (13.73)	87.08 (13.86)	86.78 (15.23)
BMI (kg/m^2) [a]	26.58 (3.33)	27.30 (3.81)	30.69 (4.36)	31.02 (4.73)
Waist circumference (cm) [a]	91.85 (9.25)	93.75 (11.12)	102.36 (10.57)	102.96 (11.43)
Hip circumference (cm) [a]	100.12 (7.41)	101.77 (8.24)	106.58 (9.43)	106.24 (9.59)
WHR [a]	0.92 (0.06)	0.92 (0.06)	0.96 (0.05)	0.97 (0.05)
Kilocalories [a]	2380.01 (696.36)	2218.81 (734.51)	2270.88 (661.07)	2297.18 (731.06)
Alcohol consumption (g/die) [a]	15.99 (15.66)	14.61 (15.96)	17.87 (16.56)	16.53 (15.86)
Wine consumption (ml/die) [a]	140.37 (144.57)	123.25 (147.42)	157.82 (154.68)	141.31 (151.01)
TG (mg/dL) [a]	113.79 (81.69)	114.34 (68.52)	168.16 (118.51)	198.54 (145.51)
TC (mg/dL) [a]	194.65 (37.18)	197.75 (36.30)	205.31 (38.95)	204.11 (40.13)
HDL-C (mg/dL) [a]	48.67 (12.06)	48.08 (11.47)	44.39 (9.91)	43.53 (10.14)
LDL-C (mg/dL) [a]	123.67 (30.78)	126.47 (32.32)	127.74 (33.81)	123.70 (36.88)
Glucose (mg/dL) [a]	107.45 (24.12)	108.93 (21.02)	113.65 (24.74)	114.59 (30.55)
ALT (U/L) [a]	17.72 (16.46)	17.35 (11.34)	21.71 (11.15)	22.36 (12.03)
Smoking habit [b]				
Never	353 (44.3)	79 (9.9)	292 (36.6)	73 (9.2)

Current	96 (41.9)	20 (8.7)	79 (34.5)	34 (14.8)
rMED [c]	8.00 (6.00-9.00)	8.00 (6.00-9.00)	8.00 (6.00-9.00)	8.00 (6.00-9.00)
Age at Death (years) [c]	62.74 (54.62-76.88)	60.82 (55.60-78.38)	65.43 (57.83-76.46)	69.76 (58.06-75.96)
Observation Time (years) [c]	17.89 (17.04-18.05)	17.91 (16.97-18.05)	17.91 (17.04-18.26)	17.95 (17.80-18.43)
Status [b]				
Alive	358 (43.8)	75 (9.2)	298 (36.4)	87 (10.6)
Dead	91 (43.8)	24 (11.5)	73 (35.1)	20 (9.6)
Job [b]				
Unemployed & Pensioner	103 (46.4)	19 (8.6)	84 (37.8)	16 (7.2)
Manager & Professional	40 (44.0)	14 (15.4)	27 (29.7)	10 (11.0)
Crafts. Agricultural and Sales Worker	180 (41.8)	36 (8.4)	165 (38.3)	50 (11.6)
Housewife	1 (100.0)	0 (0.0)	0 (0.0)	0 (0.0)
Elementary Occupation	124 (44.3)	30 (10.7)	95 (33.9)	31 (11.1)
Education [b]				
Primary School	113 (41.4)	26 (9.5)	108 (39.6)	26 (9.5)
Secondary School	155 (42.9)	43 (11.9)	128 (35.5)	35 (9.7)
High School	148 (46.1)	22 (6.9)	112 (34.9)	39 (12.1)
Graduate	33 (46.5)	8 (11.3)	23 (32.4)	7 (9.9)
Marital Status [b]				
Single	75 (53.6)	20 (14.3)	35 (25.0)	10 (7.1)
Married or Cohabiting	352 (42.0)	78 (9.3)	317 (37.8)	91 (10.9)
Separated or Divorced	13 (52.0)	1 (4.0)	11 (44.0)	0 (0.0)
Widow/er	9 (39.1)	0 (0.0)	8 (34.8)	6 (26.1)

Hypertension [b]				
No	349 (46.8)	74 (9.9)	253 (34.0)	69 (9.3)
Yes	100 (35.6)	25 (8.9)	118 (42.0)	38 (13.5)
Dyslipidemia [b]				
No	358 (47.8)	82 (10.9)	242 (32.3)	67 (8.9)
Yes	91 (32.9)	17 (6.1)	129 (46.6)	40 (14.4)
Diabetes [b]				
No	418 (45.0)	89 (9.6)	328 (35.3)	94 (10.1)
Yes	31 (32.0)	10 (10.3)	43 (44.3)	13 (13.4)

Group 1: MASLD No LTPA high and moderate; Group 2: MASLD No LTPA low and absent; Group 3: MASLD Yes LTPA high and moderate; Group 4: MASLD Yes LTPA low and absent; MASLD: Metabolic dysfunction-associated steatotic liver disease; LTPA: Leisure Time Physical Activity; SBP: Systolic Blood Pressure; DBP: Diastolic Blood Pressure; BMI: Body Mass Index; TG: Triglycerides TC: Total Cholesterol; HDL-C: High-Density Lipoprotein Cholesterol; LDL-C: Low-Density Lipoprotein Cholesterol; ALT: Alanine Amino transferase, rMED: Mediterranean relative scoring system [a] Mean (±SD); [b] Number (Percentage), [c] Median (IQR). [d]Percentages calculated for the column. Otherwise percentages calculated for the row.

Figure S1: Test of proportional- hazards assumption.

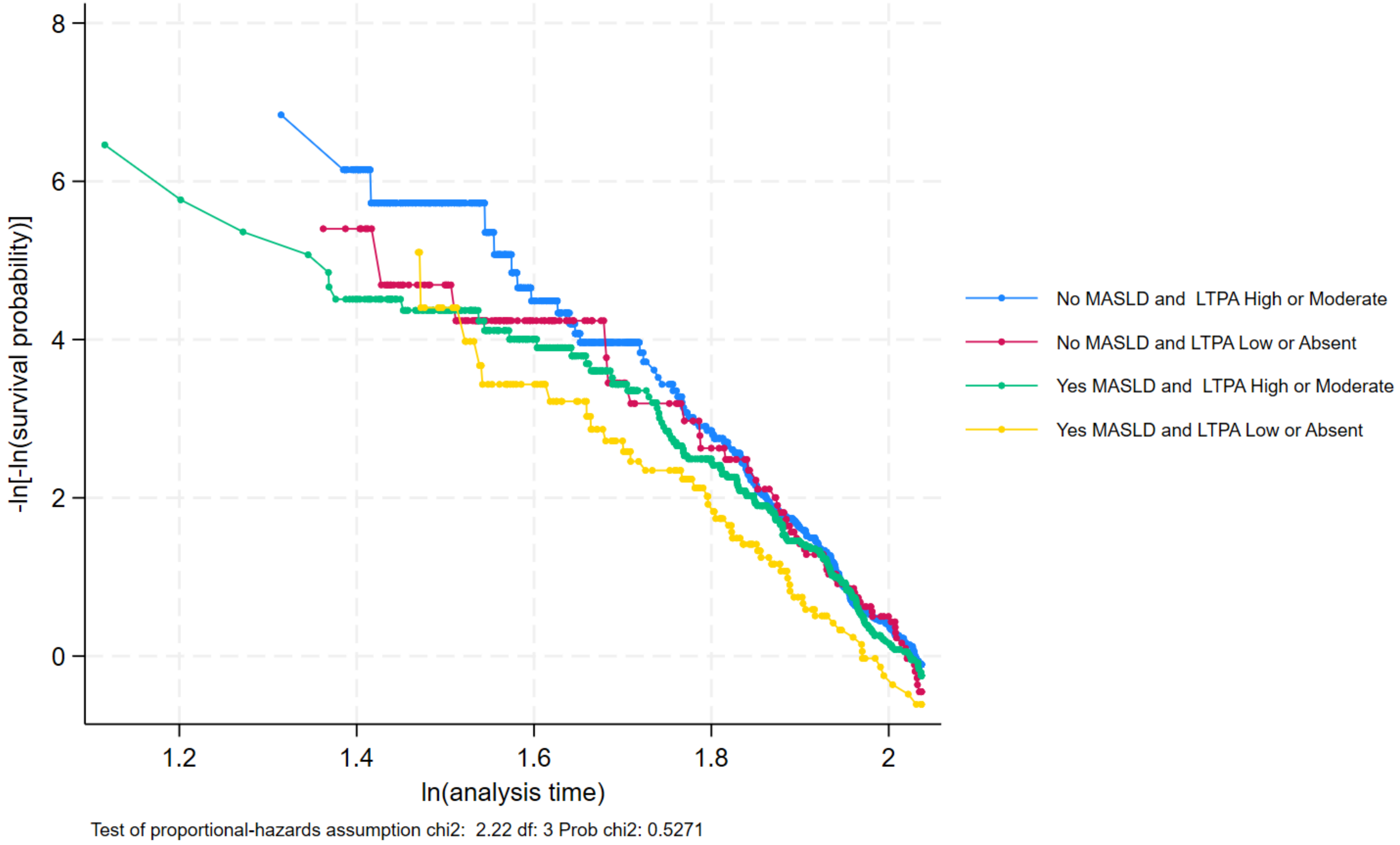

Figure S2. Lasso Cox Model

ID	Description	lambda	No. of nonzero coef.	In-sample dev. ratio	CV mean deviance
1	first lambda	**.0421165**	**0**	**0.0000**	**13.96932**
42	lambda before	**.0009287**	**12**	**0.0205**	**13.7912**
* 43	selected lambda	**.0008462**	**12**	**0.0205**	**13.79119**
44	lambda after	**.000771**	**12**	**0.0206**	**13.79121**
65	last lambda	**.0001093**	**12**	**0.0206**	**13.79202**

```
. lassocoef  minBIC adaptive, sort(coef, standardized)
```

	minBIC	adaptive
eta_50	x	x
Fumo	x	x
sexM0F1	x	x
MASLD	x	x
cate01	x	x
vino_ml_die	x	x
Education_esatto	x	x
rMED_Score	x	x
PAS	x	x
Trigl	x	x
lavoro	x	x
kilocal_day	x	x
